# Crop evapotranspiration and water productivity in Loess Plateau: A case study of Shanxi province based on SEBAL model

**DOI:** 10.1371/journal.pone.0325350

**Published:** 2025-06-04

**Authors:** Jianjun Bai, Jingshu Wang, Ping Li, Zelong Yao, Rutian Bi

**Affiliations:** College of Resources and Environment, Shanxi Agricultural University, Taigu, Shanxi, China; SMHI: Sveriges meteorologiska och hydrologiska institut, SWEDEN

## Abstract

This study focuses on Shanxi Province, located in the typical rain-fed agricultural region of the Loess Plateau, where farmland water reserves are limited and water scarcity is a significant challenge. To achieve stable and high grain yields while addressing these constraints, the primary focus must be on reducing agricultural water use while balancing productivity with water conservation, provided that sufficient irrigation sources are available. The research utilized Landsat 8 satellite data and meteorological information to simulate crop evapotranspiration (ET) in Shanxi using the SEBAL model. Based on the simulated ET values and field survey data of crop yields, the study calculated crop water productivity (WP). It then analyzed the relationship between WP and natural factors such as elevation, air temperature, and precipitation. Key findings include: Crop ET in Shanxi generally increased from northeast to southwest, with simulation results closely aligning with FAO Penman-Monteith equation calculations (relative error of no more than 5%). Crop yields ranged from 386.794 to 754.896 kg/mu, with WP varying significantly across regions—from 0.824 kg/m^3^ in the Jindongnan district to 2.532 kg/m^3^ in the Jinnan district. Regional variations in crop WP were influenced by elevation, annual temperature, and precipitation patterns. Notably, the linear correlation between WP and these factors was weaker in the Lvliang mountainous area. These findings emphasize the importance of implementing appropriate field management practices (e.g., optimized irrigation scheduling, soil moisture management) to improve both crop yields and WP.

## Introduction

Agricultural production focuses on either increasing grain yield with the same amount of water or maintaining yield while reducing water usage [1]. This efficiency is termed crop water productivity (WP) [2], which illustrates the relationship between water consumption in agriculture and crop output [3]. Improving crop WP—using less water to produce more grain—is a key goal of water-saving agricultural practices [4]. In arid and semi-arid regions, where water and grain shortages are prevalent, it is crucial to first quantitatively evaluate regional crop WP. Moreover, crop WP serves as an important metric for evaluating the effectiveness of farm irrigation management practices on crop growth. This indicator reflects the value or benefit of water used in farm management [5], making the measurement of crop WP vital for managing agricultural water and boosting production.

Evapotranspiration (ET), encompassing both soil evaporation and plant transpiration [6–8], plays a crucial role in the water cycle across various land use types and is a key component of the water and energy exchanges within the plant-soil-atmosphere continuum [9,10]. ET determines the water supply of a crop, the ability to convert more dry matter or yield under certain ET conditions, that is, WP. The SEBAL model, developed by Dutch researchers such as Bastiaanssen in the late 20th century, enables the calculation of ET using primarily remote sensing data with minimal ground meteorological data. The model’s adaptability across different spatiotemporal resolutions and its high accuracy make it a practical tool for ET estimation [11]. Raeesi et al. [12] used Landsat 8 satellite images (with the irrigation crop growing season from April 13 to October 22) and SEBAL to estimate the daily actual ET of irrigated agriculture and orchards in the Nahavand Plain. The results were compared with those of the FAO-Penman-Monteith method (RMSE = 0.82), indicating that SEBAL is efficient in estimating ET. Using the SEBAL algorithm on the Google Earth Engine (Gee) platform, Landsat images and ERA5 global reanalysis data were used to generate a time series of ET estimates (16-day intervals) for the wheat growing season from 2013 to 2022. Evaluation of the GeeSEBAL-derived data showed an RMSE of 0.98 and an MAE of 0.07. These findings highlight the importance of GeeSEBAL for estimating wheat ET in areas with limited data availability [13]. To assess the ET of the Yinchuan Plain, the Gee platform was used with Landsat TM + /OLI remote sensing images and GeeSEBAL to analyze the spatial distribution patterns of ET across different seasons from 1987 to 2020. The results indicated that GeeSEBAL is highly applicable in the Yinchuan Plain region [14]. Their findings along with those from other researchers using the SEBAL model, underscores the model’s effectiveness and widespread application in further calculating crop WP. By integrating remote sensing images, climate data, and biophysical models, the water consumption and WP of winter wheat in the clay plains of central Sudan were estimated. SEBAL and remote sensing (RS) were used to estimate the actual ET (ETa) and WP of wheat crops. The FAO Penman-Monteith method, combined with field measurements and observations, was used to validate ETa. The results show that the spatial ETa and WP maps generated by the SEBAL model and Landsat-8 imagery can improve water use efficiency at the field scale and estimate the water balance of large agricultural areas [15]. Liu et al. [16], based on high spatial resolution Landsat images from 2000 to 2020, used an improved SEBAL model to estimate the ETa of the irrigation areas in the Amu Darya Delta (IAAD) and Syr Darya Delta (IASD), and compared the WP of typical crops in both irrigation areas with statistical data. The ETa simulated by the SEBAL model showed good agreement with the crop ET calculated using the Penman-Monteith method and ground-based measurements, with correlation coefficients all above 0.7. This study provides valuable information for agricultural water resource management in the Aral Sea region. In the semi-arid irrigation maize-wheat cropping system at the ICAR-Indian Agricultural Research Institute in New Delhi, the large aperture scintillometer (LAS) was used to validate the remote sensing-based ET model Spatial Evapotranspiration Modeling Interface (SETMI). Additionally, high-resolution Sentinel-2 imagery was used at the regional scale to estimate the regional WP of wheat crops in India’s semi-arid regions. The study showed that the SETMI model provides a reliable estimate of regional wheat WP by examining its spatial and temporal variations and facilitating the creation of regional benchmark values [17]. Ghorbanpour et al. [18] used the GEE platform to calculate the actual ET for major crops in the Lake Urmia Basin, combining it with Monteith’s light utilization efficiency model to estimate yields and thereby assess crop WP. Timely and effective capture of crop ET and WP across large areas, along with the selection of appropriate field management practices to enhance crop yield and WP, is of significant importance for improving agricultural productivity and sustainability.

Shanxi Province is situated in the Loess Plateau, a region characterized by typical rain-fed and dry-farming agriculture [19,20]. The province encompasses ten water resource tertiary zones and spans two major hydrological systems: the Yellow River basin and the Haihe River basin. The province’s rivers are divided into self-originating and out-flowing types. Shanxi’s agricultural production faces several challenges, including low precipitation due to climatic conditions, a complex climate, limited land resources, underdeveloped agricultural infrastructure, low water reserves per unit area of farmland, and overall water scarcity [21,22]. Using the SEBAL model, we calculated the crop ET in Shanxi Province for the year 2020. We then determined crop WP based on this ET and actual crop yields and analyzed the correlation between crop WP and natural factors, such as elevation, air temperature, and precipitation. This study aims to provide a scientific reference for balancing high crop yields with water conservation in Shanxi Province, ensuring a reliable supply of water for agricultural production.

## Study area

**Fig 1:**
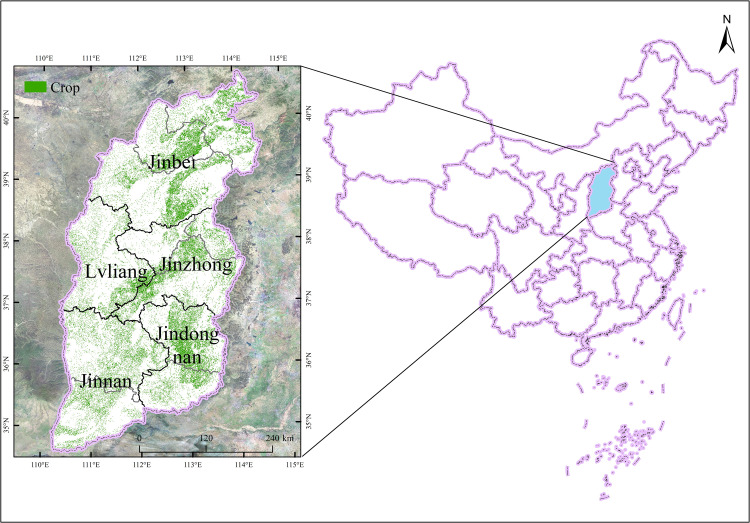
Location map of Shanxi province and crop growing areas.

Located on the eastern Loess Plateau, spanning from 34°34′N to 40°44′N and 110°14′E to 114°33′E longitude, Shanxi Province covers an area of 156,700 km^2^. It is geographically defined by the Taihang Mountains to the east, which serve as a natural barrier separating it from Hebei Province, and the Yellow River to the west and south, marking its boundaries with Shaanxi and Henan Provinces, respectively. To the north, it stretches across the Great Wall, adjoining the Inner Mongolia Autonomous Region (Fig 1). The province features complex and diverse landforms, predominantly mountains and hills, which constitute 80.1% of its area, while plains and valleys make up 19.9%. The terrain is higher in northeastern Shanxi and lower in the southwestern part. Climatically, Shanxi experiences average annual temperatures ranging from 4.2°C to 14.2°C and annual precipitation levels between 358 mm and 621 mm. Precipitation peaks from July to September, accounting for 60% to 80% of the annual total and is significantly affected by the topography.

## Data sources and research methodology

### Data sources

Landsat images offer rich spectral information and substantial data storage capacity. Notably, the thermal infrared band of Landsat is sensitive to the thermal characteristics of ground features, making these images ideal for the inverse calculation of ET used in this study. For this purpose, Landsat data were acquired from geospatial data clouds (http://www.gscloud.cn/), selecting all images covering the crop growth periods in Shanxi for the year 2020 with cloud coverage of less than 20% (path: 124–126, row:32–36). Additionally, ASTER GDEM data with a 30-m resolution were obtained from the same geospatial data clouds (http://www.gscloud.cn/). The meteorological data for this study were acquired from the China Meteorological Data Service Center (http://www.nmic.cn/), encompassing daily and monthly records of meteorological elements (including temperature, wind speed, sunshine, relative humidity, and precipitation) coinciding with satellite overpasses. Using the elevation, latitude, and longitude information from meteorological stations, these data were interpolated using ANUSPLIN software to generate meteorological grid data congruent with the size of the ET simulation pixels [23]. Ground survey data were primarily focused on various attributes including types of ground crops (such as corn, wheat, cereals, soybean, and potato), crop planting density, crop sowing and harvesting times, peak-growth stages, irrigation scales, and vegetation growth.

### SEBAL

**Fig 2:**
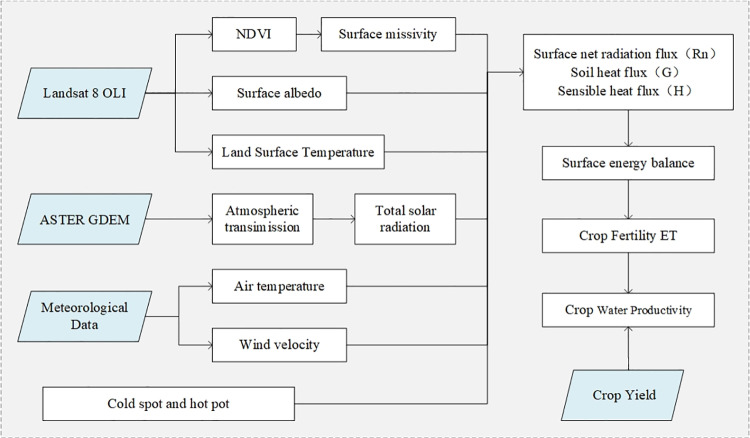
Research methodology and scientific workflow for crop ET in Loess Plateau.

The SEBAL model, introduced by Bastiaanssen in 1995, is a sophisticated energy balance model that uses the surface energy balance equation to process remote sensing image data from platforms such as Landsat [24,25]. This model enables researchers to inversely calculate various parameters such as surface albedo, NDVI, vegetation cover, surface emissivity, and surface temperature (Fig 2). Subsequently, these parameters are used to estimate the net radiation flux and soil heat flux, incorporating relevant meteorological data. The sensible heat flux is then inversely calculated using the cold and hot pixel values identified in the selected remote sensing images. This calculation facilitates the determination of the latent heat flux and daily ET [25]. In practical selection, the criteria for “cold spots” are: points with low surface temperature and high normalized vegetation index; the criteria for “hot spots” are: points with high surface temperature and low normalized vegetation index. The underlying principle of the SEBAL model is based on the energy balance at the Earth’s surface, which can be succinctly described by the following equation:


$$\lambda ET=R_{n}-G-H$$ (1)

In equation (1), Λ*ET* denotes the latent heat flux (W/m^2^); *Λ* denotes the latent heat coefficient of water vaporization (J/kg); *ET* represents the ET (mm); *R*_*n*_ signifies the net radiation flux at the Earth’s surface (W/m^2^); *G* denotes the soil heat flux (W/m^2^); and *H* denotes the sensible heat flux (W/m^2^).

The net radiation flux at the Earth’s surface represents the net balance of shortwave and longwave radiation and is the primary source of energy for surface ET. It also constitutes the main form of energy exchange between the Earth’s surface and the atmosphere, including momentum, moisture, and other material molecules. The net radiation flux is calculated using the following equation:


$$R_{n}=(1-\alpha )R_{s↓}+R_{L↓}-R_{L↑}-(1-\varepsilon )R_{L↓}$$ (2)

In equation (2), *α* denotes the surface albedo; *ε* represents the surface emissivity; *R*_*S*↓_ signifies the amount of solar shortwave radiation reaching the Earth’s surface (i.e., total radiation emitted by the sun) (W/m^2^); *R*_*L*↓_ stands for the downward atmospheric longwave radiation reaching the Earth’s surface (W/m^2^); and *R*_*L*↑_ indicates the longwave radiation emitted by the Earth’s surface (W/m^2^).

Soil heat flux refers to the energy stored in the soil and vegetation during soil heat exchange [26]. It can be expressed using the following equation:


$$G=\left[\frac{T_{s}-273.15}{\alpha }(0.0038\alpha +0.0074\alpha^{2})(1-0.98{NDVI}^{4})\right]R_{n}$$ (3)

In equation (3), *T*_*S*_ denotes the Earth’s surface temperature (K); and *NDVI* stands for the normalized difference vegetation index.

Sensible heat flux measures the energy exchange between the Earth’s surface and the atmosphere and is primarily determined by the Earth’s surface temperature, air temperature, and aerodynamic impedance [27]. It is calculated using the following formula:


$$H=\frac{\rho_{air}C_{p}d_{T}}{r_{ah}}$$ (4)

In equation (4), *ρ*_*air*_ denotes the air density (Kg/m^3^); *C*_*p*_ represents the constant-pressure specific heat of air (value: 1004J/(kg·K)); *d*_*T*_ indicates the temperature difference between the Earth’s surface and the reference height Z (generally 2 m); and *r*_*ah*_ denotes the aerodynamic impedance (s/m).

The SEBAL model calculates daily ET using the evaporation ratio method. The remote sensing data capture instantaneous ET values at the time of satellite transit. By converting these values from an instantaneous to a daily time scale, daily ET is derived from the instantaneous measurements. The calculation equation is as follows:


$$\Lambda_{inst}=\frac{R_{n}-G-H}{R_{n}-G}=\frac{\lambda_{ET}}{R_{n}-G}=\Lambda_{24}$$ (5)

In equation (5), *Λ*_*24*_ denotes the evaporation ratio within 24 h. When the evaporation ratio is known, *ET*_*24*_ is calculated using the following equation:


$${ET}_{24}=\frac{86400\Lambda_{24}R_{n24}}{\lambda }$$ (6)

In equation (6), *R*_*n24*_ denotes the daily net radiant flux at the Earth’s surface (one day comprises 86,400 s).

### Penman-Monteith

In this study, crop *ET*_*C*_ was used to verify the daily ET values inversely calculated from remote sensing data [28]. Crop ET is calculated using the following equation:


$${ET}_{C}=K_{C}\times {ET}_{0}$$ (7)

In equation (7), *K*_*C*_ denotes the dimensionless crop coefficient, and *ET*_*0*_ represents the reference crop ET (mm).

First, *ET*_*0*_, or reference crop ET, can be calculated using the FAO-56 Penman–Monteith formula [29], which was recommended by the Food and Agriculture Organization of the United Nations (FAO) in 1998:


$${ET}_{0}=\frac{0.408\Delta \left(R_{n}-G\right)+\gamma \frac{900}{T+273}U_{2}\left(e_{s}-e_{a}\right)}{\Delta +\gamma \left(1+0.34U_{2}\right)}$$ (8)

In equation (8), *ET*_*0*_ denotes the reference crop ET; *R*_*n*_ represents the net radiation flux; *G* signifies the soil heat flux; *γ* indicates the psychrometric constant; *U*_*2*_ stands for the average wind speed at a height of 2 m; *e*_*s*_ denotes the saturation vapor pressure; *e*_*a*_ represents the actual vapor pressure; *Δ* denotes the slope at the *T* position of the curve of relation between temperature and saturation vapor pressure; and *T* indicates the daily average air temperature at a height of 2 m.

### WP model

Crop WP measures the crop yield per unit of water consumption, specifically the amount of grain harvested per unit of water used during crop growth [30]. This metric is crucial for optimizing agricultural irrigation and maximizing crop yields [31]. It is calculated using the following equation:


$$WP=\frac{yield}{ET}$$ (9)

In equation (9), *yield* denotes the crop yield per unit area (kg/m^3^), and *ET* represents the crop ET.

## Results and analysis

### Spatial pattern of daily vaporization and its verification

**Fig 3:**
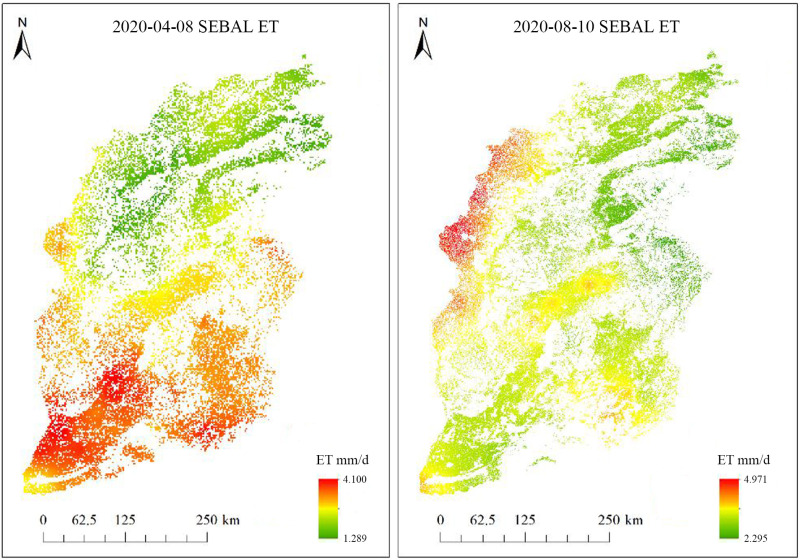
Crop growing areas in Shanxi Province on April 8 and August 10, 2020.

Fig 3 illustrates Shanxi’s daily crop ET on April 8th and August 10th, 2020, calculated using the SEBAL model. On April 8th, daily ET in the study area ranged from 1.289 to 4.100 mm/d. With rising temperatures, crop growth accelerated and entered a critical stage, necessitating substantial irrigation as crop leaves expanded rapidly, resulting in vigorous ET. By August 10th, as temperatures reached their annual peak, the average daily ET was 2.24 mm/d. During this period, summer crops were in full growth, experiencing high temperatures and significant precipitation. This led to larger crop leaves and intensive absorption of soil moisture by crop roots, culminating in heightened leaf transpiration and soil evaporation, i.e., increased overall ET. Spatially, the distribution of ET in Shanxi showed an increase from the northeast to the southwest, which aligns with the province’s topographic trend. Daily ET was typically higher in Southern Shanxi, often exceeding 3 mm/d, and reached approximately 2 mm/d in most parts of the Lvliang mountainous area.

Using meteorological data from three Shanxi stations—Mount Wutai, Taigu, and Lishi—we calculated daily ET with the Penman–Monteith formula and compared these values with the daily ET simulated by the SEBAL model, based on Landsat 8 remote sensing images, as described in Table 1. Influenced by topographic factors, the simulation results for the Mount Wutai station exhibited significant errors, while the results for the Taigu station showed only minor discrepancies. Specifically, absolute errors ranged from 0.0786 to 0.1797, and relative errors did not exceed 5%.

**Table 1:** Comparison between the calculated values and model inversion values of daily ET of Shanxi’s winter wheat.

Station	Date	Penman–Monteith	SEBAL	Absolute errors	Relative errors
Mount Wutai	20200810	3.5812	3.7609	0.1797	4.78%
Taigu	20200810	5.0843	5.1629	0.0786	1.52%
Lishi	20200810	7.3481	7.5041	0.1560	2.08%

### Shanxi’s crop yield and WP

**Fig 4:**
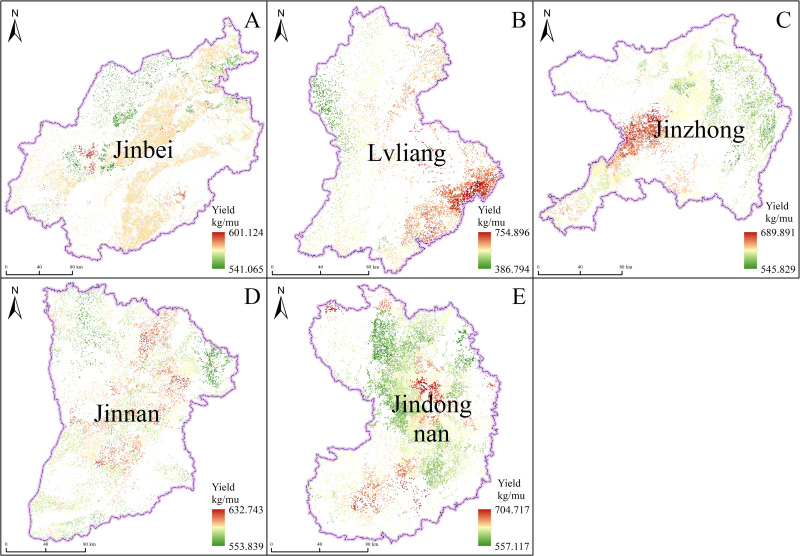
Crop yields in various regions of Shanxi province (A-D represent Jinbei, Lvliang, Jinzhong, Jinnan, and Jindongnan districts, respectively).

**Fig 5:**
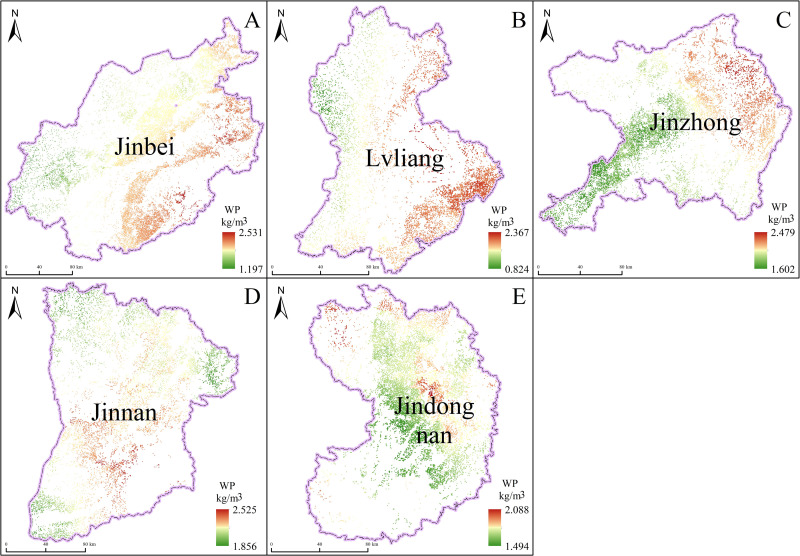
Crop WP in various regions of Shanxi Province (A-D represent Jinbei, Lvliang, Jinzhong, Jinnan, and Jindongnan districts, respectively).

Based on the inverted daily ET, we used the daily evaporation ratio interpolation method to determine monthly ET. We then combined this data with the surveyed crop yield and incorporated it into the WP equation to determine Shanxi’s crop WP, as shown in Figs 4 and 5. Shanxi was divided into five regions for this study, according to its administrative division and geographic and topographic characteristics: Jinbei district (including Datong, Shuozhou, and Xinzhou), Lvliang district (Lvliang), Jinzhong district (including Taiyuan, Jinzhong, and Yangquan), Jinnan district (including Linfen and Yuncheng), and Jindongnan district (including Jincheng and Changzhi). In Jinbei district, the average crop yield is 577.046 kg/mu, with lower yields in Shuozhou and higher yields in Datong and Xinzhou. Crop WP ranges from 1.197 to 2.531 kg/m^3^ with higher values in Xinzhou, suggesting that crop WP in Jinbei district is primarily affected by crop yields. Therefore, to increase crop WP, it is necessary to improve factors such as fertilizer application, farmland management, and the use of high-yielding varieties. In Lvliang district, average crop yields range from 553.839 to 632.743 kg/mu, and crop WP varies from 0.824 to 2.367 kg/m^3^, averaging 1.742 kg/m^3^. Areas with high crop yield and WP are mainly found in the middle reaches of the Fen River, while crop WP is relatively low in the western mountainous areas. In Jinzhong district, the average crop yield ranges from 545.829 to 689.891 kg/mu, approximately 33.835 kg/mu higher than in Jinbei district. Areas with high average crop yield are primarily in Jinzhong city. Crop WP in Jinzhong district ranges from 1.602 to 2.479 kg/m³, averaging 1.958 kg/m^3^. High crop WP is mostly found in the high-elevation eastern part, while it is relatively low in the valley plains on both sides of the Fen River. This indicates favorable water conditions but extremely high ET, resulting in low yield per unit of water consumption and relatively low water utilization efficiency.

In Jinnan district, the average crop yield is 588.069 kg/mu, with crop WP ranging from 1.856 to 2.526 kg/m^3^. High yields and WP are predominantly found in the lower reaches of the Fen River, as opposed to other counties in Jinnan district, making these areas ideal for extensive farm irrigation. In major crop-producing regions such as the Zhongtiao Mountains (in Jinnan district) and the Lvliang Mountain (in Western Shanxi), crop WP is notably low, primarily due to anthropogenic factors such as insufficient irrigation. In Jindongnan district, the maximum average crop yield reaches 704.717 kg/mu, with crop WP varying from 1.494 to 2.089 kg/m³. Areas with high crop WP are generally those with high average crop yields, indicating that factors affecting crop WP in Jindongnan district are similar to those in Jinbei Shanxi. However, the average crop WP in Jindongnan district is relatively low compared to Jinbei district, suggesting lower water utilization efficiency. Across Shanxi, crop WP spans from 0.824 kg/m^3^ in the Lvliang mountainous area to 2.531 kg/m^3^ in Jinbei and Jinnan district. The regional average varies, with the lowest average crop WP at 1.724 kg/m^3^ in Jindongnan district and the highest at 2.158 kg/m^3^ in Jinnan district. This distribution indicates that Jinnan district has the highest crop yield per unit of water consumption and the highest water utilization efficiency, while Jindongnan district has the lowest. Crop WP in Shanxi exhibits significant regional variations and is heavily influenced by natural conditions such as temperature, precipitation, topography, and soil conditions, as well as factors such as irrigation conditions, field management, and crop varieties. Overall, crop WP is lower in mountainous areas than in basins. Within Shanxi’s various regions, crop WP is closely correlated with crop yield per unit area and significantly affected by irrigation conditions; it tends to be higher in areas with greater crop yield per unit area.

### Influencing factors of crop WP

Based on 2020 data concerning crop WP and natural factors such as elevation, temperature, and precipitation, we conducted a correlation analysis to identify the natural factors most closely related to the spatial variation in crop WP. As described in Table 2, in Jinbei district, crop WP is significantly correlated with elevation, temperature, and precipitation. Specifically, it is negatively correlated with elevation and precipitation, but positively correlated with average annual temperature. Regionally, the influence of elevation, average annual temperature, and precipitation on crop WP varies. In Jinbei district, crop WP tends to increase with a decrease in elevation, an increase in average annual temperature, and a decrease in average annual precipitation. Lower elevations are associated with higher crop yields per unit area, leading to increased crop WP. Conversely, while higher average annual temperatures positively impact crop yield per unit area, increased annual precipitation tends to enhance ET and water consumption, thereby reducing water utilization efficiency and forming a negative correlation with crop WP. In the Lvliang mountainous area, crop WP also shows significant correlations with elevation, average annual temperature, and precipitation. However, the correlation coefficients are relatively low compared to other regions in Shanxi, suggesting a weaker linear relationship between these factors and crop WP. Here, crop WP increases with higher elevation, indicating more efficient water utilization in Lvliang’s dry-farming areas. An increase in average annual temperature may lead to more ET, reducing crop WP, while increased precipitation can effectively boost crop yield per unit area, thereby enhancing crop WP.

**Table 2:** Correlation between crop WP and elevation, temperature, and precipitation in various regions of Shanxi province.

	DEM	Temperature	Precipitation
Jinbei District WP	−0.326^**^	0.133^**^	−0.193^**^
Lvliang District WP	0.050^**^	−0.069^**^	0.066^**^
Jinzhong District WP	0.326^**^	−0.499^**^	−0.187^**^
Jinnan District WP	−0.392^**^	0.437^**^	−0.242^**^
Jindongnan District WP	0.226^**^	−0.485^**^	0.016

** Correlation is significant at a confidence level (two-test) of 0.01.

In Jinzhong district, crop WP shows significant correlations with elevation, temperature, and precipitation. Specifically, it is negatively correlated with average annual temperature and precipitation, but positively correlated with elevation. More specifically, crop WP tends to increase with increasing elevation, decrease with increasing average annual temperature, and increase with decreasing average annual precipitation. The rise in elevation corresponds to higher water utilization efficiency in Jinzhong district’s dry-farming mountainous areas. Additionally, increases in average annual temperature and precipitation typically lead to more ET, causing crop WP to decrease with these rising variables. In Jinnan district, crop WP is significantly correlated with elevation and average annual temperature and precipitation. It is positively correlated with average annual temperature but negatively correlated with elevation and average annual precipitation. Specifically, crop WP tends to increase with decreasing elevation, increase with the increasing average annual temperature, and increase with decreasing average annual precipitation. In Jinnan district, higher crop yield per unit area in plains contribute to greater crop WP; the increasing average annual temperature positively affects the crop yield per unit area, whereas increasing average annual precipitation tends to increase crop ET, resulting a negative correlation with crop WP. In Jindongnan district, crop WP is significantly correlated with elevation and average annual temperature (specifically, negatively correlated with average annual temperature and positively correlated with elevation), yet it shows no significant correlation with average annual precipitation. Specifically, crop WP tends to increase with the increasing elevation but decrease with the increasing average annual temperature. In Jindongnan district, the increase in elevation indicates higher water utilization efficiency in the region’s dry-farming mountainous areas, while the increase in average annual temperature may lead to higher crop ET, thereby negatively affecting crop WP.

## Discussions

In Shanxi Province, the terrain gradually slopes from the high mountain areas in the northeast to the lowland areas in the southwest. This topographical variation has a significant impact on local climate conditions [32,33], such as precipitation, temperature, and wind speed, which in turn directly affect the ET rate [34]. Higher-altitude areas typically have lower temperatures and less precipitation, while lower-lying areas experience higher temperatures and relatively more precipitation. This climatic difference leads to a gradual increase in ET from northeast to southwest. In lowland areas, soil moisture is usually richer, and water supply is abundant, leading to higher ET rates. In contrast, high-altitude areas with scarce water resources generally exhibit lower ET rates [34]. Overall, the southern part of Shanxi Province has higher ET rates compared to the north due to its warmer and more humid climate. This phenomenon is consistent with the warmer, more humid climatic conditions in the southern region. Future studies should focus on integrating a broader range of meteorological and geographic data to further validate the spatial distribution characteristics of ET and explore the specific impact of different terrains on ET. This will not only help in understanding the ET differences across different regions of Shanxi Province but also provide comparative analysis for regions with similar climatic conditions.

In mountainous areas, higher altitudes and lower temperatures limit crop WP due to the shorter growing seasons and limited water supply [35]. In contrast, lowland areas, with higher temperatures and relatively more precipitation, can provide more abundant water sources, supporting higher WP [4]. The spatial differences in WP are not only closely related to the seasonal variations in temperature and precipitation but also to topographical factors. Mountainous and hilly terrains generally lead to faster water drainage, which reduces the effective storage of moisture in the soil and limits the improvement of WP. On the other hand, flat areas tend to retain water more easily, providing more abundant soil moisture, which promotes the effective use of water by crops [4]. Additionally, the seasonal distribution of WP is closely related to precipitation patterns [35]. Concentrated seasonal precipitation can cause water shortages during dry periods, affecting water use efficiency, while evenly distributed rainfall helps ensure a stable water supply, enhancing WP. In arid or semi-arid regions, insufficient precipitation typically requires irrigation to supplement the water supply, which is crucial for improving WP. Excessive precipitation may lead to poor soil drainage, affecting root growth and thereby reducing crop WP.

Compared to arid or semi-arid regions with similar climatic conditions, the relationship between WP and climate change in Shanxi Province shows certain similarities [36]. For example, in the arid regions of Inner Mongolia and Gansu [37,38], the higher temperatures and low precipitation generally result in low crop WP. In these areas, factors such as infertile land and uneven spatial and temporal distribution of precipitation severely impact water use efficiency. In some areas of Xinjiang, despite the low rainfall, crop WP is relatively high due to the adoption of advanced irrigation technologies and water resource management. This situation is similar to that in some regions of Shanxi Province, indicating that modern water management technologies play an important role in improving WP in areas with water scarcity. By comparing with other regions, the findings of this study can provide more references for areas with similar climatic conditions. Water scarcity is often a significant issue in these regions, but by introducing advanced irrigation technologies, rational water resource management, and adaptive analysis of topographical differences, crop WP and water use efficiency can be effectively enhanced.

## Conclusions

Firstly, the spatial distribution of ET demonstrates a clear increasing trend from northeast to southwest, consistent with the province’s topographic features. This pattern highlights the importance of terrain in shaping hydrological processes in the region. Specifically, daily ET values reached upwards of 3 mm/d in the Jinnan district, reflecting higher agricultural water demand in this area compared to most regions in Jinbei district, where ET remained below 2 mm/d. The robust validation using FAO-56 Penman–Monteith formula confirmed the reliability of remote sensing-derived ET values, with absolute errors ranging from 0.0786 to 0.1797 and relative errors consistently under 5%. This level of accuracy underscores the potential of integrating remote sensing technology for large-scale ET monitoring in Shanxi.

Secondly, the analysis of crop yields and WP reveals significant spatial disparities across the province. Crop yields per unit area vary from 386.794 to 754.896 kg/mu, while WP spans from 0.824 to 2.532 kg/m^3^. Notably, Jindongnan district exhibits the lowest average WP at 1.724 kg/m^3^, indicating suboptimal water use efficiency and lower agricultural productivity per unit of water compared to other regions. In contrast, Jinnan district demonstrates the highest WP at 2.158 kg/m^3^, highlighting its superior water use efficiency and suggesting it as a model for sustainable irrigation practices in Shanxi.

Thirdly, the relationship between elevation, temperature, precipitation, and crop WP exhibits considerable spatial variation across the province, underscoring the need for localized agricultural strategies. While these factors play significant roles in shaping WP in most regions, the findings from the Lvliang mountains stand out as an exception. In this mountainous region, WP shows relatively low correlation with elevation, temperature, and precipitation, suggesting a unique set of drivers influencing WP here. This spatial heterogeneity highlights the complexity of hydrological processes and calls for tailored management approaches in different ecological zones.
